# Involvement in cyberbullying events and empathy are related to emotional responses to simulated social pain tasks

**DOI:** 10.1177/20552076241253085

**Published:** 2024-05-15

**Authors:** Rosalba Morese, Matteo Angelo Fabris, Claudio Longobardi, Davide Marengo

**Affiliations:** 1 213307 Department of Psychology, University of Turin, Turin, Italy; 2 Faculty of Communication, Culture and Society, 27216 Università della Svizzera italiana, Lugano, Switzerland; 3 Faculty of Biomedical Sciences, 27216 Università della Svizzera italiana, Lugano, Switzerland

**Keywords:** Cyberbullying, empathy, social pain, cyberball, pain, adolescents

## Abstract

**Introduction:**

This study aims to explore the relationship between cyberbullying involvement either as a perpetrator or a victim and emotional responses to virtual social exclusion and inclusion. Previous research has predominantly focused on the impacts of in-person bullying. Our study shifts this focus to the cyber realm.

**Methods:**

A total of 156 adolescents living in northern Italy were recruited (*M*_age_: 12.26; *SD *= 0.87; 43% female). After completing measures of empathy and involvement in cyberbullying, adolescents participated in the cyberball tasks. Latent profile analysis was used to identify subgroups.

**Results:**

We found three groups: Class 3, reporting negative responses to the social exclusion tasks and positive responses to the social inclusion tasks; Class 1, reporting neutral emotional responses to social inclusion and negative emotional responses to social exclusion; and Class 2, showing neutral responses to ‘social exclusion’ tasks and strongly positive responses to ‘social inclusion’ tasks. Linear regression revealed that cyberbullies report a typical emotional response to exclusion and inclusion tasks (Class 3), whereas cybervictims are more likely to report negative responses to both exclusion and inclusion events (Class 1). High levels of empathy are associated with the manifestation of a typical emotional response (Class 3), in contrast to an impaired emotional response characterized by neutral or positive responses to conditions of ‘social exclusion’ and positive responses to conditions of ‘social inclusion’ (Class 2).

**Conclusion:**

Results underscore the complex interplay between cyberbullying roles and emotional responses to virtual social experiences. Theoretical implications and limitations of the research are discussed.

## Introduction

### Cyberbullying and psychological adjustment in adolescents

Peer victimization in adolescence is still considered a social emergency in Western populations, and among the forms of peer victimization, bullying seems to be the most studied.^1–6^ Bullying is defined as an aggressive act (physical, verbal, or social) committed repeatedly by one or more peers to cause harm to one or more victims.^7^ With the advent of new technologies, the dynamics of bullying began to affect adolescent relationships in online contexts, resulting in what has become known as cyberbullying.^8–11^ Although traditional bullying (i.e. bullying that occurs in real-world contexts rather than online) is considered more prevalent than cyberbullying,^12^ several data suggest that cyberbullying among youth has increased in recent years.^13^

Cyberbullying seems to be associated with more negative developmental outcomes for victimized youth, poorer academic performance,^14^ greater psychological maladjustment,^10,15–17^ and greater peer isolation.^18^ However, much research seems to be necessary to better understand which mechanisms are recruited in the adjustment processes of victimized adolescents. In this sense, one process implicated in the relationship between cyber victimization and negative developmental outcomes may be the proneness to interpret social information negatively.

### Peer victimization and the social information processing model

Consistent with the “social information processing model,” ^19,20^ it is possible that individuals who experienced forms of victimization are more likely to believe that they will be victimized again in the future in interpersonal relationships, and that victimized individuals are more likely to interpret the intentions of others negatively. This may also be true for children and adolescents, as some studies seem to support.^21–23^ These studies indicate that victimized children, compared to children who were not involved in peer victimization, tended to interpret others’ intentions as more malicious when they had to interpret ambiguous vignettes. In addition, some researchers^21–23^ found that victimization is associated with processes of social cognition as it relates to as is the cognitive and emotional evaluation of the experience. For example, Camodeca and Goossens^21^ found that victimized children feel more anger and sadness than nonvictimized children in ambiguous conditions. Overall, the relationship between peer victimization and social cognition appears to support what has been termed the “prevention hypothesis.” This hypothesis states that peer victimization may be related to a more negative social cognitive style that leads individuals to perceive peers more negatively, focus on threatening social causes, attribute a more dangerous and hostile character to the intentions of others, and develop a strong sensitivity to rejection.

More recently, some authors have attempted to study the relationship between prior experiences of bullying and emotional responses to potentially painful social stimuli using the experimental cyberball paradigm rather than relying on vignettes.

### Social exclusion as social pain: the cyberball task

The cyberball task is the most commonly used task for experimentally studying social exclusion and inclusion.^24^^,^^25–30^ In the cyberball game, people recruited for the study are made to believe that it is an online game in which they are the other two participants. There is an initial inclusion phase in which players pass the ball to each other and an exclusion phase in which the individual observes the other players passing the ball to each other without their own participation and reports negative feelings about the experience.

The painful experience of social exclusion can be considered a form of bullying. Social exclusion is typically associated with greater psychological distress^31^ and has been linked to recruitment of the common brain systems recruited in the experience of physical pain.^28^ For this reason, the experience of social exclusion is defined as “social pain”: the negative experience of social exclusion seems to stem from a basic human need, namely the need to belong.^32,33^ This need is basic for all humans, but seems to be particularly pronounced during adolescence.^11^ Social exclusion is considered one of the most common forms of victimization among adolescents both in the world and online, and several studies show a link between the experience of social exclusion and increased psychological distress among adolescents.^1,10,11,34,35^

### Peer victimization and response to social exclusion

Studies that have used the cyberball paradigm typically show that youth who have experienced bullying or forms of peer victimization are more sensitive to a single episode of social exclusion during the cyberball task, tend to have more negative emotional responses to social exclusion,^5,36–38^ and report higher physiological reactivity.^5,39^ For example, a study^40^ examined the neural response to social exclusion in adolescents who had experienced peer victimization. Participants underwent functional magnetic resonance imaging n) while playing a virtual ball-tossing game and were either included in or excluded from the game.

Findings indicated that participants who had experienced peer victimization showed a stronger brain response to social exclusion related to pain and negative affect. This suggests that peer victimization may lead to hypersensitivity to social exclusion and increased emotional responses to negative social events, increasing sensitivity to negative social cues.^36,40^ Thus, these children reported more negative emotional responses to both the inclusion and exclusion episodes, suggesting that victimized children tend to develop negatively biased social cognition.

### Bullies and response to social exclusion

Cyberball research has typically focused on the figure of the victim, and only a few studies have been conducted on the bully. We know that aggressive children and adolescents also tend to interpret ambiguous situations in a malicious and hostile manner,^41^ but the literature seems to point to poorer physiological activation of the bully prior to social stressors.^5^ In the experimental cyberall study, early pubertal bullies reported poor responsiveness to social exclusion.^5^ This likely reflects the lower fear of social exclusion characteristic of bullies, who may lack emotional and social ties to peers. However, other experimental results^16^ showed that bullies who experienced exclusion during the experiment experienced activation of those brain areas associated with reward learning, salience monitoring, and motivational processes. According to the authors,^16^ this finding suggests that bullying is associated with increased neural activation when a situation, in which social hierarchy cues are salient, is observed. According to the authors, the fact that previous research has found hypoactivation of these brain areas does not indicate an actual empathy deficit, but rather a difficulty in automatically processing pain in others. Therefore, exposing them to experimental conditions, in which bullying victims can connect to the emotional experiences of the other who is ostracized or bullied, would reduce hypoactivity.^16,42^

### Bullying involvement and response to social exclusion: the role of empathy

In general, it is important to better understand the relationship between involvement in bullying and emotional responses to socially painful stimuli, such as those encountered in the cyberball task. In addition, we must increase our knowledge of the possible factors involved.

Empathy may play a role in this process. Empathy, which is defined as the ability to understand and feel another's emotional state, including cognitive and affective elements, plays a central role in this mechanism.^43^ Emotions are important for processing social information because they help individuals code and interpret situations and organize their behavior accordingly.^19^ Empathy is closely related to processes of emotion regulation and prosocial behavior, and several experimental studies suggest that observing someone's social exclusion elicits the same emotional reactions and a similar neurological response as if we were excluded ourselves. Experimental research using the cyberball paradigm indicated that higher levels of empathy elicit a stronger prosocial behavior in witnesses of social exclusion.^44,45^ For example, they found that youth with higher empathy skills and lower involvement in bullying engaged in more compensatory behaviors aimed at increasing the involvement of the most socially excluded person in the game.^45^ Thus, empathic skills may be central to the emotional response to cyberball, enabling a less biased response characterized by a negative reaction to social exclusion and a positive reaction to social inclusion. Some evidence suggests that among children and adolescents, involvement in bullying, either as a victim or as a bully, tends to be associated with lower prosocial behavior and empathic skills.^46^ Specifically, it appears that bullies exhibit lower levels of affective empathy, meaning that while they can understand what others are feeling, they are less likely to empathize with what others are feeling emotionally. In contrast, bullying victims appear to have good affective empathy, but report greater difficulty understanding the mental states of other children. This is a limitation given that empathic skills play a central role in protecting children from the distress associated with peer victimization and fostering better interpersonal relationships.^46^ Although there is evidence that social exclusion, as a form of social pain, is associated with lower levels of empathy,^18,47,48^ studies that have examined the association between social exclusion and empathy using the cyberball paradigm have yielded mixed results.^48–51^ However, in these studies, which had mostly been conducted on adults, the aim was to understand experimentally whether social exclusion can make individuals more sensitive to the experience of their own or others’ suffering or whether it contributes to a decrease in empathic sensitivity. To date, however, none of the studies have examined whether the level of empathy is related to more positive or negative responses to cyberball-induced social inclusion or exclusion.

Our research aims to extend current knowledge on the relationship between cyberbullying (both victimization and perpetration) and emotional responses to social exclusion by drawing on the experimental paradigm of cyberball. In particular, considering that previous research has mainly focused on traditional bullying, our study aims to make a new contribution regarding involvement in cyberbullying both as cybervictims and cyberbullying perpetrators. Indeed, cyberbullying is a phenomenon that is becoming increasingly common among adolescents^13^ and seems to have an impact on the psychological well-being of the adolescents involved.^42^ In addition, social exclusion in the online world is a fairly common form of victimization among adolescents, and several lines of evidence suggest that peer exclusion or ostracism in the online world may be associated with increased psychological stress^35^ and lead to a physiological stress response,^52^ similar to the real world. Finally, we focused on early adolescence because this is a critical developmental period for building personal identity, and adaptation processes during this developmental period can be a protective factor for later ages. Finally, adolescents are using social media more and more independently and are increasingly oriented toward their peer group as the main social reference.^53^

### Aim of the present study

This study aims to explore the relationship between cyberbullying involvement either as a perpetrator or a victim and emotional responses to virtual social exclusion and inclusion. Previous research has predominantly focused on the impacts of in-person bullying. Our study shifts this focus to the cyber realm. Utilizing a modified cyberball experiment, we aim to determine if adolescents’ involvement in cyberbullying perpetration and cybervictimization is associated with distinct emotional reactions to virtual social inclusion and exclusion events. Our primary hypothesis posits that adolescents engaged in cyberbullying and cybervictimization will exhibit altered emotional reactions during these virtual tasks, differing significantly from those not undergoing such experiences. Specifically, we anticipate variations in the intensity and nature of their emotional responses to events of social exclusion and inclusion in a virtual context.

Furthermore, this study delves into the role of empathy in shaping these emotional responses. We hypothesize that higher levels of empathy among adolescents will predict a more emotionally congruent response pattern: more positive reactions to inclusion and more negatively charged reactions to exclusion scenarios in the virtual environment.

Additionally, we aim to examine potential gender differences in the links between cyberbullying experiences, empathy, and emotional responses to the tasks. In this case, our hypothesis is more exploratory, although we anticipate identifying distinct emotional response patterns to cyberbullying experiences between male and female adolescents, contributing to a more nuanced understanding of how gender may influence the emotional impact of cyberbullying. In exploring these hypotheses, we will control the concomitant effect of age.

## Method

### Sample

This cross-sectional, observational study was conducted by collecting data on a sample of 156 early adolescents attending middle schools in Northwest Italy, 43% of whom are females with a mean age of 12.26 (*SD *= 0.87). The sample was recruited from three public middle schools. First, we invited 10 schools to participate in the study by randomly selecting them from the official list of public schools in the region. Of these 10 schools, only three responded to the invitation. All schools reached out to for the study, encompassing both those that participated and those that did not, were situated within the same geographic region of Piedmont and characterized by a mix of medium-sized urban areas and smaller rural towns. The final sample of schools, including one from a large center and two from smaller centers, reflects a wide range of area types within this region. This variety helps reduce concerns about selection bias in the research. Additionally, these schools had similar demographic profiles in terms of the student populations they serve. However, it is important to note that due to a relatively low rate of participation, the sample obtained should be seen as a convenience sample. Therefore, caution should be exercised when considering the extent to which these findings can be generalized to broader Italian contexts.

The students comprising the sample expressed their interest in voluntarily participating in the study, the details of which were communicated in a letter to the students and their families. The students were able to take part in the study if they were native Italian speakers and had not been diagnosed with any mental disabilities or deficits that could prevent them from participating in the study. In our sample, all students owned a personal smartphone and regularly accessed the internet. After obtaining approval from the respective school administrators, the research project was introduced to both guardians and pupils. Exclusive enrollment encompassed solely those students who procured written informed consent from their parents or legal guardians. We also obtained written informed consent from the students themselves. Monetary compensation for participation was categorically absent. The participants first completed the self-report measures anonymously and were then subjected to the cyberball task a week later.

All participants were duly apprised of the essence and objectives of the research in full alignment with the ethical guidelines stipulated by the Italian Association for Psychology (AIP). The procedural protocol identified as number 290961 garnered the requisite endorsement from the Institutional Review Board affiliated with the authors’ academic institution. Stringent adherence was maintained to the ethical directives set forth by the Italian Society of Psychology.

### Instruments

#### Cyberbullying and cybervictimization

Participants’ involvement in cyberbullying and cybervictimization was assessed using a 16-item scale evaluating four different forms of cyberbullying.^54^ The instrument includes four subscales, each consisting of four items, namely, cyberbullying (e.g. “I excluded someone from an online group to make him/her feel bad”), cybervictimization (e.g. “ Some of my embarrassing pictures or images were spread without my permission”), defending (e.g. “I was included in an online group to make fun of someone, but I defended him/her”), and passive bystander (e.g. “I was aware that someone was threatened or insulted via the phone or the Internet and I did nothing”). Participants rated the frequency of each event on a five-point scale, including the following response categories: 1 — never; 2 — once during the last month; 3 — two or three times during the last month; 4 — once a week; and 5 — more than two times a week. Note that in the present study, only the cyberbullying and cybervictimization subscales were administered. Item responses were averaged to create a total score for each subscale (cyberbullying: *mean *= 1.13, *SD *= 0.47; cyberbullying: *mean *= 1.38, *SD *= 0.56). Based on Cronbach's alpha, the score reliability was acceptable (cyberbullying: α = 0.68; cybervictimization: α = 0.64)

#### Empathy

We administered the Italian version of the Interpersonal Reactivity Index (IRI)^55^ 28-item version. The item assessed two broad components of empathy, namely, cognitive empathy (perspective taking and fantasy subscales, each comprising seven items) and affective empathy (empathetic concern and personal distress subscales, each comprising seven items). Some example items are as follows: “After seeing a play or movie, I have felt as though I were one of the characters” (fantasy); “I sometimes find it difficult to see things from the “other guy's” point of view” (reverse item) (perspective taking); “I often have tender, concerned feelings for people less fortunate than me” (empathetic concern); and “In emergency situations, I feel apprehensive and ill-at-ease” (personal distress). Items were rated on a five-point Likert scale from 1 (never true of me) to 5 (always true of me). The IRI has shown good reliability and validity in early adolescent and adult samples.^56^ In keeping with previous studies, to ensure high reliability, item responses were averaged to create a total empathy score (mean = 3.17, *SD* = 0.47). Based on Cronbach's alpha, the score reliability was good (α = 0.80).

#### Cyberball task

To evoke the experience of social inclusion and exclusion, we employed the widely used game of cyberball.^30^ In particular, we used a modified version developed by Novembre et al.^24^ and subsequently used by Morese et al.,^25^ which does not use simple cartoons, but virtual videos of real people. The task was structured in 10 total blocks that provide two conditions: the (a) “social inclusion” condition and the (b) “social exclusion” condition. Into each block, the game of the ball throw involves a total of 12 steps. In 10 blocks, the experimental condition of social inclusion is administered in which the participant gets to receive the ball. In the other 10 blocks, the condition of social exclusion was administered, in which the participant received less than a third of the total steps (for details on stimuli and procedure, see Novembre et al.^24^). At the end of each block, each participant was expected to report the valence (positive or negative) of the emotions felt during the conditions of social inclusion and exclusion and the intensity on a Likert scale (from −4 to +4).

### Data analysis

First, the emotional response data collected by administering the cyberball game to evoke social inclusion and social exclusion conditions were analyzed using the latent profile analysis (LPA) approach. Note that participants’ responses to the cyberball tasks were collected in the form of ordinal item responses to a nine-point response scale ranging from −4 ‘very negative’ to +4 ‘very positive.’ Because of a relatively extended response range (nine points), we treated response data as continuous. On these data, we run a series of LPAs using a mixture modeling approach. Note that the cyberball indicators failed to comply with normality based on the Shapiro–Wilk test. To deal with non-normality, estimation was performed via maximum likelihood with robust standard errors (MLR), which is robust to deviation from normality in the indicator set.^57^ We estimated four models each based on k = 2 to k = 5 classes. To identify the model that provided the best fit between model-fit and model-complexity, we used the Lo–Mendell–Rubin (LMR) statistics to define the optimal number of classes.^58^ When comparing competing models with k and k − 1 classes, a non-significant test (*p* > .05) indicates that the model with k − 1 classes is to be preferred (Lo et al. 2001). Additionally, the Bayesian information criterion (BIC), entropy statistic, average classification probabilities, and class counts were used to compare competing models. We preferred models reporting lower BIC values (Schwartz, 1978), higher entropy values, and higher average classification probabilities, with values ≥.80 a stronger statistical separation between classes and greater predictive power.^59^ We also preferred LPA solutions that are based on class counts and identified classes consisting of large groups of participants, as opposed to small groups, including <30 individuals. Note that an a-priori power analysis for the LPA was not performed because of a lack of knowledge of population parameter values, which are required when performing Monte Carlo simulations for power analysis in LPA.^60,61^ Note that at N = 156, while our sample sits at the lower end of the continuum of sample sizes emerging from reviews on the actual use of LPA in social sciences^61–63^, it sits above the limit of 100 participants noted as sufficient for appropriate applications of LPA.^64^ These analyses were performed using Mplus version 8.

For each experimental session of the cyberball task, the one-way analysis of variance (ANOVA) was then applied to elaborate the significance of mean differences in participants’ emotional responses across the LPA classes, with Bonferroni post-hoc tests for determining pairwise comparisons (*p* < .05). Finally, we looked at the association between adolescents’ involvement in cybervictimization and cyberbullying, as well their empathy level, in predicting their emotional profile when responding to the cyberball task (i.e. simulated social inclusion and exclusion tasks). Additionally, we explored gender as a moderator of these effects. For the purpose of these analyses, we used a nominal regression approach controlling for age and gender. Analyses were performed in two steps: in Step 1, main effects (i.e. cybervictimization, cyberbullying, and empathy) and control variables (age and gender) were tested as predictors of the LPA classification; and in Step 2, interaction effects between gender and cybervictimization, cyberbullying, and empathy were additionally included in the model. To ensure the robustness of findings, regression analyses were elaborated with a bootstrap approach (1000 samples). These analyses were run in SPSS version 23.

## Results

### Latent profile analysis of emotional responses to simulated social inclusion and social exclusion

LPA models with one to five classes were compared to define the optimal number of classes. The BIC and LMR statistics for the estimated models are shown in Table 1. Note that the LMR statistic failed to reach significance when comparing the three and four class models (LMR = *p* = .17), indicating the three-class model as the one providing a better fit to the data. In contrast, the four-class solution reported the lowest BIC value. To choose between the three- and four-class models, we looked at entropy values, posterior class probabilities, and class counts. Overall, the models showed very similar entropy (three-class model: *entropy *= 0.86; four-class model: *entropy *= 0.87) and posterior class probability values (three-class model: range of posterior probability = 0.92–0.94; four-class model: range of posterior probability = 0.89–0.97). In turn, the four-class model included at least one class with a count <30, while all classes in the three-class model included a class count ≥30. Combining the results of the LMR test and this information, we decided to select the three-class model as the preferable solution.

**Table 1.** LMR tests, BIC and entropy statistics for the estimated LPA models.

Classes	Parameters	LMR	p	Entropy	BIC
1	20	—	—	—	5719.13
2	31	245.68	0.00	0.85	5524.58
3	42	135.07	0.03	0.86	5442.63
4	53	103.91	0.18	0.87	5392.40
5	64	49.56	0.91	0.87	5397.50

**Figure 1.**
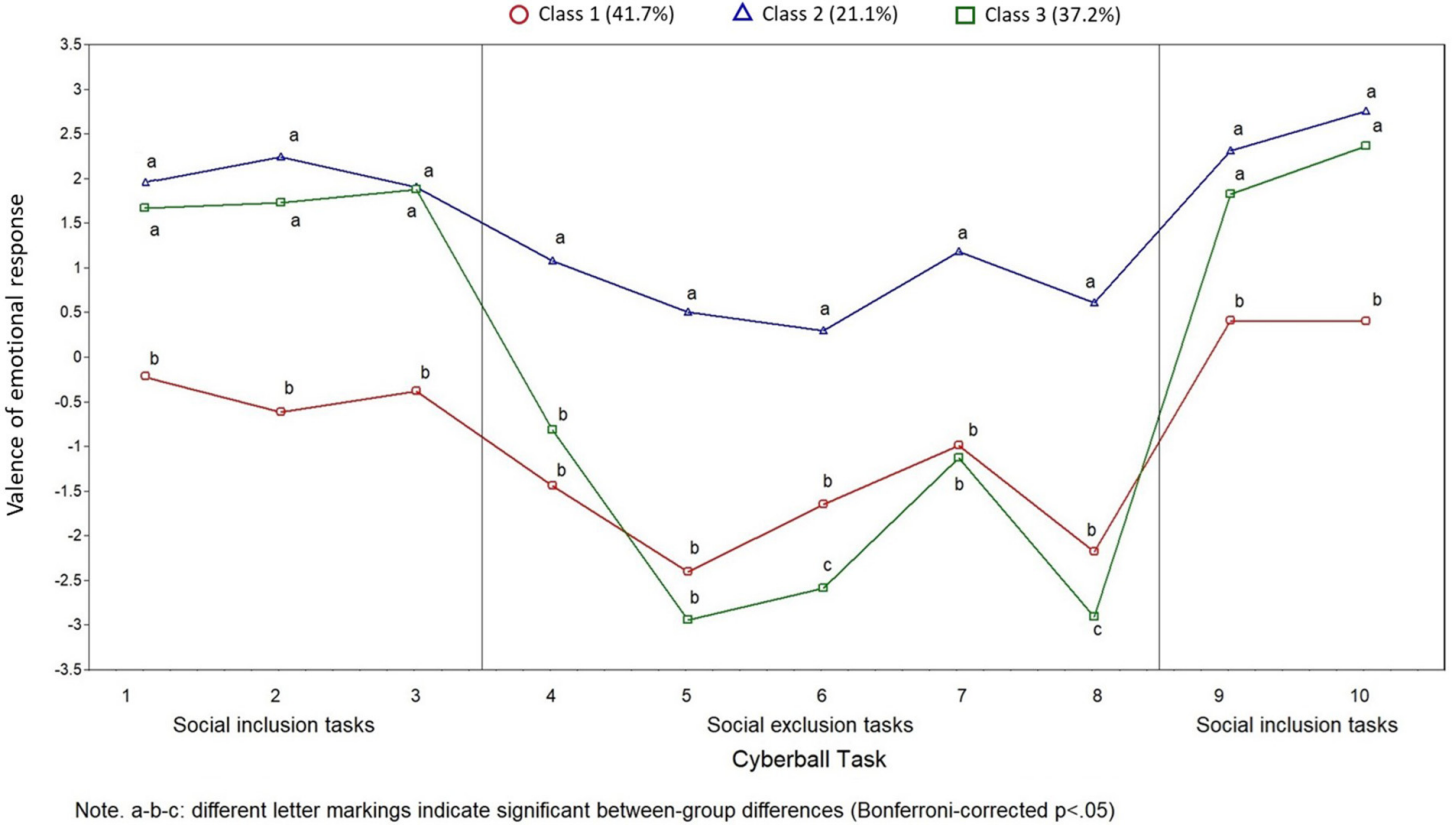
Latent profile analyses of the emotional responses simulated social inclusion and social exclusion.

Figure 1 provides a visualization of the emotional scores for the 10 cyberball tasks in the three classes. Note that tasks 4 to 8 represented “social exclusion” tasks, while tasks 1–3, 9, and 10 were “social inclusion” tasks. In Figure 1, for each task, post-hoc results of the one-way ANOVA investigating differences in emotional responses across classes are shown with a compact letter display (CLD) approach, wherein classes showing significant mean differences are labeled with different letters (i.e. a and b) and classes showing no significant differences are labeled with the same letter. Looking at Figure 1, it is easy to see that Class 1 (n = 65, red line) includes individuals that, on average, show a specific emotional response profile with neutral emotional responses to the “social inclusion” tasks and negative emotional responses to the “social exclusion” tasks. In turn, Class 2 (n = 33, blue line) includes individuals who show neutral to slightly positive responses to “social exclusion” tasks and decidedly positive responses to “social inclusion” tasks. Based on ANOVA analyses, classes 1 and 2 showed average differences in responses to all tasks, with Class 1 showing a more negative profile between the two classes. Finally, Class 3 (n = 58, green line) shows an intermediate profile with highly positive responses to “social inclusion” tasks and either moderately or highly negative responses to “social exclusion tasks. Based on ANOVA analyses, classes 3 and 2 show similarly positive responses to “social inclusion” tasks, while Class 1 deviates from both these classes in showing significantly more negative responses to “social inclusion” tasks. In turn, Class 3 shows significantly more negative responses to “social exclusion” tasks than Class 2, while both classes 1 and 3 show a similarly negative profile on the “social exclusion” tasks, with only minor differences.

### The role of adolescents’ involvement in cyberbullying events and empathy in emotional responses to simulated social tasks

Next, we explored the role of adolescents’ involvement in cyberbullying events, which was measured by assessing both their cybervictimization and cyberbullying, as well as their empathy level, with relevance to predicting the three-class categorization of responses to simulated social inclusion and social exclusion tasks obtained by performing the LPA analyses on the cyberball data. Because the outcome is a class variable (i.e. a categorical variable), analyses were performed using multinomial regression approach and using one of the classes as a reference. For the purpose of this analysis, Class 3 was used as a reference group because individuals in this class showed an emotional response that was compatible with a simple ostracism effect (i.e. a negative response to social exclusion stimuli and a positive response to social inclusion stimuli).^30^ For a meta-analysis, see Hartgerink et al.^65^ Table 2 reports the results of multinomial regression analyses. Results of Step 1 of analyses indicated that cybervictimization was positively related to the likelihood of belonging to Class 1, as opposed to Class 3 (reference group), that is, to show a more overly negative response to inclusion stimuli, but a similar profile on social exclusion stimuli (Figure 1). Cyberbullying showed an opposite effect, indicating a lower likelihood of belonging to Class 1, as opposed to belonging to Class 3 (reference group). That is, cyberbullies might not show the heightened negative responses to social inclusion events seen in Class 1 possibly because their role as perpetrators of bullying does not align with the more victim-centric emotional profile of Class 1.

**Table 2.** Multinomial regression: adolescents’ involvement in cyberbullying events and empathy as predictors of the classification of emotional responses to social tasks.

	LPA class (Reference: Class 3)					
	Class 1			Class 2		
	B	p	Exp(B)	B	p	Exp(B)
Step 1: Nagelkerke R2 = 0.190						
Gender	−0.348	0.428	0.706	0.306	0.554	1.357
Age	0.199	0.355	1.221	−0.330	0.226	0.719
Cybervictimization	0.594	0.033	1.811	−0.062	0.847	0.940
Cyberbullying	−0.890	0.022	0.410	−0.336	0.400	0.715
Empathy	−0.088	0.686	0.916	−0.525	0.029	0.592
Step 2: Nagelkerke R2 = .236						
Gender	−0.216	0.637	0.614	0.190	0.783	0.725
Age	0.271	0.234	0.205	−0.328	0.238	0.198
Cybervictimization	0.923	0.008	0.013	−0.149	0.777	0.754
Cyberbullying	−1.628	0.001	0.002	−0.114	0.748	0.757
Empathy	−0.186	0.522	0.509	−0.467	0.207	0.223
Cybervictimization × Gender	−0.512	0.416	0.365	0.161	0.858	0.810
Cyberbullying × Gender	1.570	0.012	0.024	−0.390	0.585	0.577
Empathy × Gender	0.219	0.631	0.607	−0.091	0.866	0.865

Note: Gender is coded: female = 1; male = 0. Bootstrap p values are reported (1000 samples).

**Figure 2.**
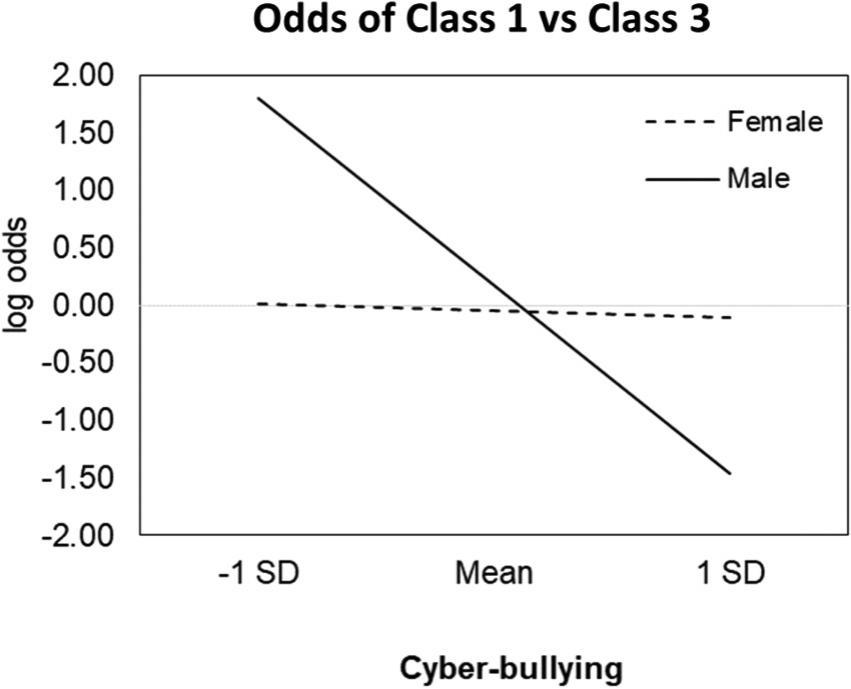
Graphical representation of interactions between cyberbullying and gender in predicting Class 1 (Reference group: Class 3).

Still regarding Step 1 of the analyses, we found that empathy predicted a decrease in likelihood of belonging to Class 2, as opposed to Class 3 (reference group), that is, empathy appears to decrease the odds that participants will show a positive (as opposed to negative) response to exclusion stimuli while showing a similar positive response on the social inclusion stimuli (Figure 1). Finally, Step 2 of the analysis only revealed a significant interaction effect, namely, a positive interaction effect between gender and cyberbullying in predicting the likelihood of belonging to Class 1, as opposed to belonging to Class 3 (reference group). The interaction effect is visualized in Figure 2, suggesting that the aforementioned effect of cyberbullying on class membership (i.e. lower likelihood of belonging to Class 1, as opposed to belonging to Class 3) might be only observed for males.

## Discussion

The main aim of this study was to investigate the influence of previous experiences of cyberbullying and cybervictimization on emotional responses to simulated events of social inclusion and social exclusion. To achieve this goal, we recruited a sample of adolescents and collected data on their recent involvement in cyberbullying events both as perpetrators and victims. In addition, participants played a modified version of the well-known cyberball game,^24,25^ and their emotional responses to conditions of social inclusion and social exclusion were recorded using self-report measures.

The participants’ responses to the modified cyberball game were examined using the LPA approach, which led us to identify three groups with different patterns of emotional responses. One of the groups (Class 3) exhibited an emotional response characterized by a negative reaction to the experience of social exclusion and a positive reaction to the experience of social inclusion, consistent with a simple ostracism effect.^30^ A second group (Class 1) exhibited a peculiar emotional response profile characterized by neutral emotional responses to the “social inclusion” tasks and negative emotional responses to the “social exclusion” tasks. A third group (Class 2) was characterized by neutral or slightly positive responses to the “social exclusion” tasks and markedly positive responses to the “social inclusion” tasks.

In line with our first hypothesis, previous experiences in cybervictimization and cyberbullying showed a significant impact on the participants’ responses to social inclusion and exclusion tasks. The results of the regression analysis showed that participants who personally reported having been victimized online at least once in the last month (i.e. cybervictimization) tended to deviate from a typical emotional response (Class 3) and instead tended to report neutral emotional responses to the “social inclusion” tasks and negative emotional responses to the “social exclusion” tasks (Class 1). This finding is consistent with the previous findings of Lansu et al.,^36^ who suggested that children victimized by peers may have negatively biased social cognition, leading them to not experience positive emotions or to experience outright negative emotions, even during social inclusion events. Our data thus appear to add to the literature and suggest that cyberbullying is an aversive experience that may affect adolescents’ social cognitive style, consistent with previous findings on traditional bullying, in particular, they are consistent with neuroscience research highlighting that adolescents’ brains are highly sensitive to the state of social exclusion in a social group.^39,66–68^ Fuhrmann et al.^66^ demonstrated the effects of social exclusion using the cyberball game on the cognitive performance of working memory in adolescence. In contrast, participants reporting acting as a bully when online (i.e. cyberbullying) were less likely to show this altered, more overly negative emotional response (Class 1) and instead show a typical emotional response (Class 3), although this effect was only supported for male participants. Thus, our data seem to indicate that cyberbullies report emotional responses to social exclusion in a typical way (i.e. a negative response to social exclusion condition and a more positive response to social inclusion condition). It is possible that cyberbullies are less affected by involvement in bullying in terms of social-cognitive style. Therefore, cyberbullies may preserve a greater ability of online victimized individuals to respond to painful social stimuli, such as social exclusion, with a typical response partner. Although the literature suggests a deficit in empathic skills in cyberbullies, we should keep in mind that some authors point out that, from a physiological perspective, the experience of social exclusion also seems to activate adolescents who victimize peers. Thus, our data seem to suggest in cyberbullies a more typical response and a probably lower impact of involvement in cyberbullying on the development of social cognition.

In line with our second hypothesis, we found that high empathy levels were associated with showing a typical emotional response (Class 3), as opposed to an altered emotional response characterized by neutral or positive responses to “social exclusion” conditions, and overly positive responses to “social inclusion” conditions (Class 2). Empathy is central to an individual's interpersonal functioning and plays a central role in the processes of emotion regulation and prosocial behavior.

Several experimental studies have shown that high levels of empathy lead to prosocial and defensive behavior in bystanders.^44,45^ According to the authors, the bystander may be more inclined to empathize with the suffering of the victimized peer and refine various defensive or intervention strategies based on the perceived emotions.^44^ Our data reported that high levels of empathy are associated with a more typical response to the experience of social exclusion triggered by the cyberball task. It is possible, therefore, that empathy and social exclusion may play an important role in processing social information,^67^ and this may be particularly relevant for victimized youth who tend to experience and develop more negative feelings and expectations toward others and toward the quality of others’ intentions. High levels of empathy are reflected in higher levels of emotional reasoning and perspective-taking skills. In this sense, emotion recognition and empathic responsiveness play an important role in encoding and interpreting social cues.^20^ Overall, then, higher levels of empathy may be related to a more typical response pattern in the cyberball game, indicating a greater ability to interpret and perceive socially painful stimuli in these subjects while likely representing a lower risk for cognitive biases. Note that while high levels of empathy were associated with more typical emotional responses to social exclusion, the absence of significant gender differences in this link is noteworthy. This could suggest that irrespective of gender, empathy plays a crucial role in how adolescents respond emotionally to social inclusion and exclusion.

## Limitation and future direction

Our results seem to indicate that cyberbullies show more typical responses than victims of cyberbullying. They may suggest several interpretations that can be explored in future investigation. It would be important and interesting to develop new research directions and perspectives that further investigate these neurophysiological and psychological aspects, such as those of hypersensitivity, in order to better identify other risk factors, such that new intervention models can be implemented. In addition, other limitations may come into play. For example, the sample is limited and not representative of the population. New directions could, therefore, invest in future research that can capture larger and more representative samples. In addition, it might be interesting to replicate the study in different cultural contexts to explore possible differences according to cultural context. Finally, participants in our study completed self-report measures of their involvement in cyberbullying and empathy. This could increase the bias related to text comprehension or social desirability. Future studies might, therefore, resort to other survey instruments or external observers.

## Conclusions

In summary, our study contributes to the current literature by demonstrating that participants who are victims of cyberbullying, but not cyberbullies, tend to report a change in responses to the cyberball game. In this sense, victims of cyberbullying tend to respond more negatively to both inclusion and exclusion experiences, suggesting a perception of stimuli characterized by a negative cognitive bias. Thus, cybervictimization may be associated with the internalization of negative expectations about others and a reduction in the ability to experience positive emotions, even in more positive situations, as in the case of the social inclusion induced by the cyberball experiment task. In addition, our data seem to suggest that empathy is a factor associated with emotional responses to social stimuli, particularly social exclusion and inclusion. It appears that higher levels of empathy tend to be associated with more typical responses to social exclusion and social inclusion, which are associated with negative and positive emotional responses, respectively. Higher empathy competence may influence social information processing more appropriately and adaptively.
